# Budgeting of non-commercial clinical trials: development of a budget tool by a public funding agency

**DOI:** 10.1186/s13063-019-3900-8

**Published:** 2019-12-11

**Authors:** Hilde Nevens, Jillian Harrison, France Vrijens, Leen Verleye, Nelle Stocquart, Elisabeth Marynen, Frank Hulstaert

**Affiliations:** Belgian Healthcare Knowledge Centre – KCE, Kruidtuinlaan 55, 1000 Brussel, Belgium

**Keywords:** Clinical trials, Public funding, Non-commercial, Clinical trial costs, Resource use, Cost drivers, pragmatic

## Abstract

**Background:**

Investigator-led multicentre randomised trials are essential to generate evidence on the optimal use of medical interventions. These non-commercial trials are often hampered by underfunding, which may lead to difficulties in gathering a team with the necessary expertise, a delayed trial start, slow recruitment and even early trial discontinuation. As a new public funder of pragmatic clinical trials, the KCE Trials programme was committed to correctly pay all trial activities in order to assure timely delivery of high-quality trial results. As no appropriate trial budget tool was readily publicly available that took into account the costs for the sponsor as well as the costs for participating sites, we developed a tool to make the budgeting of a clinical trial efficient, transparent and fair across applicants.

**Methods:**

All trial-related activities of the sponsor and sites were categorised, and cost drivers were identified. All elements were included in a spreadsheet tool allowing the sponsor team to calculate in detail the various activities of a clinical trial and to appreciate the budget impact of specific cost drivers, e.g. a delay in recruitment. Hourly fees by role were adapted from published data. Fixed amounts per activity were developed when appropriate.

**Results:**

This publicly available tool has already been used for 17 trials funded since the start of the KCE Trials programme in 2016, and it continues to be used and improved. This budget tool is used together with additional risk-reducing measures such as a multistep selection process with advance payments, a recruitment feasibility check by sponsor and funder, a close monitoring of study progress and a milestone-based payment schedule with the last payment made when the manuscript is submitted.

**Conclusions:**

The budget tool helps the KCE Trials programme to answer relevant research questions in a timely way, within budget and with high quality, a necessary condition to achieve impact of this programme for patients, clinical practice and healthcare payers.

## Background

Randomised controlled trials (RCTs) are essential to generate evidence on the safety, efficacy and effectiveness of medical interventions [1–3]. Public funding is needed to answer important clinical questions for which the prospect of financial profit for industry is very limited, such as comparative effectiveness, drug repurposing research and research on surgical techniques, psychotherapy, physical therapy, diets, etc. [1, 4].

Trials are most informative for healthcare decision-makers if they are practice-oriented; these are also known as pragmatic trials. Such trials recruit a broad patient population, focus on patient-relevant outcomes and include usual care as a comparator [5–7]. Post-market practice-oriented trials funded with public money remain a main source of comparative effectiveness data. These trials have proven to be practice-changing in areas ranging from neonatology [8] to oncology [9, 10] and may even lead to regulatory actions, as seen after the 6S trial [11].

In addition to the benefit to patients, funding clinical trials with public money may have a positive return on investment [1, 8, 12]. European funders of clinical trials aiming for efficiency gains [1] include the National Institute for Health Research (NIHR) in England [13, 14], the Netherlands Organisation for Health Research and Development (ZonMw) [15] and, more recently, the Belgian Healthcare Knowledge Centre (KCE) [16]. The KCE Trials programme has funded multicentre comparative effectiveness trials since 2016 and has a yearly budget of €10 million.

Most hospitals are used to evaluate trial budgets for on-site activities proposed by industry. These fees often constitute a net source of income for the hospital. For England and Australia, such site-related costs have been made public [17, 18]. However, when it comes to trial sponsor activities, hospitals and academic groups may have less expertise in preparing a budget for trial costs [19]. In part, this may be caused by the limited funding available for such research. Lack of funding is a frequently reported issue for investigator-led clinical trials, and thus voluntary activities are required from all parties [20, 21].

Clinical trials are assumed to be expensive, and efforts are underway to make them more efficient, e.g. through the direct use of routinely collected data in registries [22] or electronic health records [23]. The reported average cost was US$12 million for 28 phase 3 trials funded by the National Institutes of Health (NIH) in the field of neurology [24]. The reported median cost for a pharmaceutical industry phase 3 trial was US$21.4 million (2010–2015 data from seven major pharmaceutical companies) [25]. Another publication on pharmaceutical trials in the USA (2004–2012 data) reported that the average cost of a confirmatory trial (phase 3) ranged from US$11.5 million (dermatology) to US$52.9 million (pain and anaesthesia) [26, 27]. The number and types of clinical procedures involved were the key drivers of direct costs (accounting for 15–22% of the total), followed by administrative staff costs (11–29% of the total) and site monitoring costs (9–14% of the total).

Publications detailing the complete cost structure of a clinical trial remain very scarce. In preparation of a retrospective analysis of the costs of two trials [28], a systematic review on the topic did not identify a single publication detailing the costs and resources used for the various clinical trial activities [29]. Speich et al. recently conducted a systematic scoping review with the goal to identify publicly available budget tools for randomised controlled trials (RCTs) [30]. A total of 25 tools were identified, and 7 of those could be used for planning an entire budget. Most tools were simple spreadsheets without any further information on how they were developed or proper guidance for use. A modified version of the KCE Trials budget tool, used for the first international KCE-ZonMw Belgium-Netherlands Funding of International Trials (BeNeFIT) call, was included in this review.

In this paper we present the 4th version of a tool to structure the costs of the activities needed to conduct a high-quality, multicentre practice-oriented clinical trial. This tool is used by applicants to apply for funding from KCE, and is publicly available for download on the KCE website (see 2019 version on https://kce.fgov.be/en/kce-trials-2019-investigator-led-call). The aim is to ensure a correct, fair and consistent budget per task across the trials funded, while still maintaining the flexibility to adapt each budget depending on the main cost drivers and specificities of each trial [16].

## Methods

To calculate the overall cost of the trial, we categorised a list of study-related items and tasks that are considered necessary for conducting a clinical trial according to the International Conference on Harmonisation (ICH)-Good Clinical Practice (GCP) guidelines. To standardise as much as possible, the costs are calculated using formulae linked to pre-defined study and budget parameters. Any study-specific modifications of the pre-defined items or formulae should be justified.

As the main cost drivers of a clinical trial, we included the planned numbers of study subjects (screened, randomised, completed), study sites, protocol-related visits per subject, study monitors, on-site and off-site monitoring visits, the expected maximum number of suspected unexpected serious adverse reactions (SUSARs—typically a low number in comparative effectiveness trials but can be higher in repurposing trials) and the trial complexity. In addition, the planned dates are to be entered for first patient in (FPI), last patient in (LPI), last patient last visit (LPLV), database lock (DBL) and clinical study report (CSR), which contractually in the KCE Trials programme should be available within 6 months after LPLV. The duration of the trial (defined as FPI to CSR) will have a major impact on the need for study personnel, and this will highly influence the costs. A realistic estimation of the timelines is hence of importance.

Table 1 shows the main cost drivers and their influence on the sponsor study budget. Instead of discussing the formulae used for calculating the budget, we illustrated the impact of specific variables on the overall cost using an easy-to-read table. The interested reader can find all formulae in the publicly available budget tool.

**Table 1:** Influence of main cost drivers on the study budget

Sections in study budget	Main cost drivers of study budget				
	Recruitment period (FPI to LPI)	Study period (FPI to CSR)	No. of patients	No. of study visits	No. of sites
Project design and set-up					++
Regulatory and ethics review	+				++
Monitoring	+	+	+	+	+++
Quality assurance					
Trial master file handling & administration	+	+			+
Safety			+		
Data management		+	+		
Statistics, report and publication					
Project management	+	+++			++
Patient and public involvement		+			
Intervention (medication/device) handling		+	+	+	+
Site costs			+++	+	+
External vendors/contractors/central review					

+ low impact, ++ moderate impact, +++ high impact

A separate table lists the standard hourly fees for the different roles involved in the study. The rates assume 1600 productive hours per year for personnel, based on published Belgian data [31]. The rates are adjusted for inflation, based on an average seniority level and do not include any overhead costs. They were benchmarked with publicly available salaries of academic personnel and with rates received from the human resources department of Belgian university hospitals, applicable in 2019 (see Additional file 1: Table S1).

Where possible, we opted to work with fixed amounts. This is the case for, e.g. protocol writing or statistical analysis. The fixed amounts are based on an estimation of the time investment by different people involved in a task; we avoid lengthy discussions on how much time is needed for a certain task. In the first version of the budget tool, hourly fees for all items and tasks were used. However, after a small number of budget negotiations, we decided to set a fixed fee for as many items as possible to pursue a standard approach across the projects. For example, a lump sum of 40 000 euros is foreseen for the development of a full protocol and tasks related to study set-up, including statistical and health economic support. This was originally based on 90 hours of work for the chief investigator, 275 hours for the project manager, 40 hours of statistician time and 40 hours for a health economist, as was suggested in the first version of the tool. However, as some budget proposals received largely exceeded the suggested time investment without proper justification, we decided to go for a fixed amount. This is, however, not possible for all tasks, especially the ones that are dependent on the duration of the trial. For those tasks, both the default number of hours as well as the role with a fixed hourly rate are pre-populated in the worksheet. Where justified, the complexity of the study and the setting may slightly increase or decrease the time involvement or cost of each task. It is assumed that each specific task is performed by appropriately qualified staff, not overqualified or underqualified individuals. For example, it is not considered appropriate to give a junior PhD student the role of project manager for a multicentre randomised trial or to have a project manager perform the time-consuming duties of an administrative assistant.

As the information available in the public domain on the sponsor cost of a trial is limited to clinical trial units in the UK [32], we based our sponsor costs on the past real-life experience of the authors: together we have about 100 years of experience in managing clinical trials in the biopharmaceutical industry, a contract research organisation (CRO), hospitals and an academic sponsor organisation. In addition, the initial tool was fine-tuned based on consultancy provided by an experienced former director of a CRO (BV, see Acknowledgements). We benchmarked our figures with trial budget proposals received from commercial and non-commercial CROs. Feedback obtained from applicants selected for funding is used to further improve the tool. Currently, version 4.0 is in use.

A margin of 15% is added to study personnel costs to account for higher seniority, future increases in inflation, unforeseen delays and extra costs or capacity needs during the study. Another reason for adding this margin was to support the learning curve of the Belgian trial centres in the first years of the KCE Trials programme. For the fixed amounts, the margin is already included. In general, overhead costs of 17% may be added to personnel costs and should include office costs including standard information technology items at the sponsor and the sites.

In case a specific task is outsourced to a third party or for the purchasing of study-specific equipment, medication or devices, a tender procedure may be needed.

## Results

The publicly available budget tool is a spreadsheet with an accompanying guidance document. The tool consists of different tabs detailing the input variables related to the trial characteristics and the personnel costs, the calculated overall costs for the sponsor and the site costs. Extra tabs can be used if needed, for example to detail the costs of a subcontractor.

### List of funded trial activities

The activities in a clinical trial and their costs can be grouped into larger categories, as listed and discussed in the following subsections. Table 2 illustrates the use of the tool with a practical example of a trial. For personnel-related cost items, 15% margin and 17% overhead are included. An additional table shows a more elaborate example of the results of a budget calculation with the tool (see Additional file 1: Table S2).

**Table 2:** Simplified example based on the use of the budget tool (version 4.0)

Project design and set-up	€84,825
- Development of budget and study protocol (including amendments, consent form and any questionnaires), risk assessment and management plan, contracting with funder and vendors, initial selection of sites/GPs - On-site feasibility check (per site, for 50% of the sites) - Remote feasibility check (for other sites) - Negotiation of site agreements - Subcontracting with tender	
Regulatory authorities, ethics committees, insurance	€29,835
- Submission to the leading ethics committee - Submission to the ethics committees of participating sites - Communication with ethics committee per study year - Submission to regulatory authorities - Communication with regulatory authorities per study year	
Study monitoring	€266,079
- Development of a monitoring plan, risk-based - Study monitor familiarisation with trial - On-site initiation visit, monitoring visits, closure visit - Fixed average transportation fee per on-site visit - Remote monitoring visits	
Quality assurance	€7020
- Sponsor audit on site 2 days by independent auditor; includes visit, preparation and report	
Trial master file (TMF) & administration	€66,234
- Set-up of (electronic) TMF - Set-up of investigator files, 4 h of clinical trial assistant per site - Maintaining files, 12 h of clinical trial assistant per site per year - Sponsor making payments to the sites, 1 per site per year - Archiving, 2 large boxes plus 1 per 4 sites for 25 years	
Safety monitoring and expedited reporting	€14,040
- SUSAR documentation and expedited reporting - Data safety monitoring board (DSMB) meeting (1x/year)	
Data management	€141,570
- Data management plan, database and electronic case report form (eCRF) set-up for comparative effectiveness trial - Import, validation, coding, query management per patient (2 h per patient)	
Statistics, report and publication	€58,500
- Statistical analysis plan, randomisation plan, programming - Statistical analysis and clinical study report - Publication, including open access fee	
Project management	€367,497
- Trial management group meeting (2x/month until end of recruitment, monthly thereafter) - Trial steering committee meeting (3x/year during first year, then 2x/year) - Investigators meeting, at study start and end - Project manager time per year: 0.2 full-time equivalent (FTE) + 0.025 FTE per site	
Patient and public involvement	€6552
- Participation during study design and set-up phase - Participation of 2 representatives in steering committee meeting	
Study intervention/investigational medicinal product (IMP) handling	€152,340
- Purchase of intervention/IMP/placebo, blinding, packaging, labelling, recovery and destruction of unused product - Storage and distribution - Central randomisation system and unblinding if applicable	
Site costs	€518,310
- Start-up fee - Per patient visit 2 h study nurse and 10 min clinician time + 30 min clinician time at first visit - Lab sample whole blood at 5 visits for central lab - Local pharmacy costs for transfer study - Archiving box for 25 years	
External vendors	€0
- Study-specific equipment or external services are already included in the IMP budget	
TOTAL BUDGET without value-added tax (VAT)	€1,725,672
TOTAL BUDGET with 21% VAT	€2,088,063

To calculate the budget for this study, we took into account the following study parameters: 20 sites, 400 patients, 7 visits per patient, recruitment period of 18 months, treatment period of 24 months, total study duration of 4 years, 5 on-site monitoring visits and every 2 months a remote monitoring visit, 3rd party placebo development, packaging, labelling, distribution

#### Project design and set-up

A fixed amount is foreseen for the set-up of the trial. This includes the development of the full protocol and the risk assessment plan and development and translation of informed consent forms and any other documents for patients (leaflets, questionnaires, etc.). It also covers the costs related to the set-up of the budget and the negotiation of the contract with the funder. If there is a cost for the use of specific questionnaires, the licence fees can be added in this section.

In order to mitigate the risk for the funder, the sponsor and the participating sites, trial feasibility visits are conducted during the trial preparation phase. Both the availability of patients and the suitability of the sites are checked. This type of visit is standard practice in industry-driven trials but is often lacking in non-commercial trials due to budget constraints. On-site feasibility checks are typically conducted by the sponsor for about half of the sites and for a representative sample in the case of general practice (GP) trials. A fixed amount is paid to the sponsor and includes the preparation of the visit, the arrangement of the appointment with the different parties involved, travel time, discussions on site and time for report writing. Other sites have a remote feasibility assessment done by the sponsor. In addition and not included in the budget tool, an independent CRO is directly paid by the trial funder to conduct a feasibility check of the recruitment before the funding contract is signed. In practice, these site visits are often prepared and performed together with the sponsor, thus creating a learning environment.

The negotiation of contracts between the funder, the sponsor and the sites proves to be a time-consuming activity. This is reflected in the budget tool, whereby this activity is more costly for trials conducted in a complex hospital setting compared with trials in general practices. Also, for subcontracting to third parties a fee is foreseen, which is higher in the case of a public tender procedure.

#### Regulatory authorities, ethics committees, insurance

In Belgium, each clinical trial is currently reviewed by a central ethics committee and the ethics committee of each participating hospital. A fixed fee is foreseen for the work involved in the submission to the leading central ethics committee and a smaller fee for each submission to a site ethics committee. In addition, a dossier is to be submitted to the regulatory authorities for trials involving medicinal products and trials with medical devices that are not CE-marked or not used for their intended purpose. A fixed fee is foreseen to prepare the dossier. In Belgium, non-commercial trials obtain a waiver for the review fees of ethics committees and regulatory authorities, but this differs by country.

Additionally, a yearly fee is foreseen to meet the reporting requirements to ethics committees and regulatory authorities.

The implementation of European Union (EU) Clinical Trials Regulation 536/2014 will have an impact on this section, as there will be a single submission through the EU portal, and therefore a separate submission for ethics (central and local ethics committees) will no longer be applicable for interventional trials with an investigational medicinal product (IMP). Other type of trials (non-IMP) may require a different submission process, which may vary from country to country.

The cost of the mandatory trial insurance can be added separately to the budget but is often a moderate amount in large hospitals running multiple trials. This item might be different depending on the insurance requirements in each specific country, e.g. no-fault liability insurance in Belgium.

#### Trial monitoring

A fixed amount is paid for the development of a risk-based monitoring plan. The training of the monitors on the trial protocol and the related procedures is added as a separate item.

For each site initiation visit, study monitoring visit and study closure visit, up to 16 h of monitor time is foreseen, depending on the study complexity. This time allows for preparation, travel time, visit and reporting. For trials in a general practice setting, a maximum of 8 h is used. Compensation for transportation costs is also added per visit. The travel time in Belgium is estimated to be between 2 and 4 h, which might need to be increased when planning for monitoring visits in countries with a wider geographical spread of the investigational sites.

Remote monitoring should be planned according to the risk-based monitoring plan. Every 2 months 2 to 4 h per site are typically planned for this activity. This may vary by trial complexity and number of patients.

#### Audit

A fixed amount is paid to the sponsor, allowing them to perform an audit of a participating site, using either an in-house auditor or an external consultant. The amount includes outsourcing costs, audit preparation, a 2-day on-site audit, the report and the follow-up.

#### Administration and filing

A fixed amount is paid to set up the trial master file. In addition, generally 4 h of clinical trial assistant time is paid per site to prepare an investigator site file. Typically, a total of 12 h of clinical trial assistant time per site per year of the trial is paid to maintain the files.

As sponsor support, 25 years of archiving of study documents are foreseen, i.e. 2 boxes for the sponsor and 1 box for every 4 sites.

A fixed amount is paid to the sponsor for the calculation of payments made to each site once or twice a year after confirmation of completeness of the data by the monitor and the data manager.

#### Expedited safety reporting and data safety monitoring board

For most pragmatic trials with medicines or devices used for their intended purpose and having a well-documented safety profile, only suspected unexpected serious adverse events may require expedited reporting. This item should be addressed in the risk assessment plan and in the relevant sections of the protocol. For each expedited report a fixed amount is foreseen. If justified based on the risk analysis, data safety monitoring board (DSMB) meetings are budgeted using a fixed amount per meeting. This includes the upfront statistical analysis in preparation of the meeting. Note that in the case where no DSMB is judged necessary, the trial steering committee, included under the project management, should monitor the progress of the trial including any safety issues.

#### Data management

The number of data items collected in a pragmatic trial should be kept to a minimum and should only address the research questions in the protocol. A strong focus on the collection of the essential data items will have an impact on the time needed for data entry, query generation and resolution and data management in general. Electronic case report forms (eCRFs) are preferred to capture the data.

Based on past experience and quotes from commercial providers, there is a fixed amount of €75,000 for data management of pragmatic low-complexity trials and a higher fee of €125,000 for high-complexity trials such as phase 3 repurposing trials. This amount includes all costs for the data management plan, the design and hosting of the eCRF, the clinical trial database and a separate safety database if needed, any importation of data, reconciliation of the safety database (if applicable) and database lock (DBL).

In addition, 2 to 4 h of data manager time per patient (depending on trial complexity and trial duration) is planned for data coding, programming of queries to be sent in batches to sites, query follow-up and resolution. If the study runs for more than 4 years with multiple visits, an additional budget may be required for data management activities.

#### Statistics, study report and publication

A fixed amount is foreseen to cover all costs associated with statistical input in the trial design, the statistical analysis plan, the programming and conduct of the analyses, the writing of the study report and the publication of the main study findings. The publication of the main trial results should be accessible free of charge (open access), and any costs of publication are included in the fee.

#### Project management

A multicentre trial needs a dedicated project manager. The time allocated to the study will depend on the trial complexity, the number of sites and patients involved, the recruitment speed, etc. The formula for this task takes into account the duration from first patient in (FPI) to the study report. Any time invested by the project manager prior to FPI is accounted for in the amounts foreseen for project design and ethics committee submission.

There is a dedicated line in the budget tool for project management, but the time invested by the project manager is also taken into account in some fixed amounts, e.g. for the different types of meetings, the site payment fee calculation and report and publication writing.

A meeting of the trial management team followed by a status report for KCE is expected every 2 weeks during recruitment and once monthly thereafter and is paid with a fixed amount per meeting. It includes the time invested by the core team members including any preparation and report writing after the meeting.

KCE requires the presence of a trial steering committee that should include among others the chief investigator, the project manager, the study statistician, at least one independent subject matter expert, two patient representatives and at least one investigator of a participating site. Trial steering committee meetings (three in the first year and two per subsequent year) are budgeted at a fixed price that includes costs for preparation, meeting room provision, reporting and covering the expense notes of participants.

Investigator meetings are budgeted at a fixed price at study start and study end. However, additional investigator meetings may be justified if the trial is conducted in a large number of sites that are geographically spread over the country.

The basic project manager time, not included in the preceding items, is typically estimated at 0.2 full-time equivalent (FTE), to be increased to 0.3 for more complex trials or large GP trials. To this estimated figure, 0.025 FTE per hospital site or per 4 GP sites is added. A trial involving 20 hospitals would thus receive a budget for 0.7 additional project manager FTE.

#### Patient involvement

A fixed amount is foreseen for documented involvement of patients or their representatives at the design stage of the trial. In addition, continued involvement is supported by covering the costs for two patient representatives to attend each trial steering committee meeting.

#### Study intervention/investigational medicinal product

The budget required for the purchase of the intervention, the IMP or placebo, the blinding, packaging, labelling and distribution to the sites will vary largely by study, and a public tender may be needed. For open-label trials with interventions that are reimbursed under the health insurance system, it is encouraged to evaluate the possibility of routine distribution and reimbursement systems, thus avoiding the risk of double payment and cost for additional labelling.

In addition to the production of any placebo, one needs to take into account the timelines and budget for stability testing, blinding, packaging, labelling and distribution of study medication to the sites. This activity can be outsourced. Marketing authorisation holders may or may not want to provide the active product and placebo free of charge or at a discount. Commercial companies that provide products or devices without charge or at a reduced rate will need to acknowledge the terms and conditions of the funding agreement between KCE and the sponsor.

Additional cost items may include the services for central good manufacturing practices and a central system for randomisation and unblinding, if not already included in the eCRF system.

#### Overview of site costs

The start-up costs for a site include contracting, team composition, familiarisation with the trial and time spent during site initiation. Different amounts have been defined for trials in hospital and GP settings. Costs for study initiation at the pharmacy or local lab are to be added if applicable. Pharmacy costs should take into account the different activities related to clinical trials that are carried out by the pharmacy personnel, e.g. local storage, distribution, recovery and destruction.

As the trials try to be as pragmatic as can be justified, most activities may reflect routine care. Therefore, the time spent by site personnel will be limited to data entry, simple questionnaires and oversight of this activity. A default of 2 h of study nurse time is estimated per study visit, plus 10 min of investigator time. For the visit where the informed consent needs to be obtained, an additional amount to cover the costs for the informed consent discussion is suggested. Additional time needed for specific investigations can be inserted in the budget tool. However, reference to protocol requirements that are not standard of care is needed to justify costs on a case-by-case basis.

#### External vendors/contractors and central review

All costs related to the outsourcing of activities that do not relate to any of the preceding activities (e.g. central review of images, etc.) should be included in this section. This section should also include costs for any specific equipment that needs to be purchased for the study. A public tender procedure may apply.

### Payment schedule

Significant efforts are expected from the applicant team to prepare a full proposal for a multicentre trial. Therefore, KCE pays a non-refundable advance of €12,500 to cover these costs. In addition, an advance of €12,500 may be requested by the candidate sponsor to perform a site feasibility check. The aim is to maximally reduce the trial risk for the applicant, the sites and the funder before the protocol and funding contract are finalised [16].

Once the budget is finalised and approved, the payment schedule is developed. In the KCE Trials programme, a fee-for-performance principle is applied for payment to sites and sponsor. Typically 20% is paid at contract signature, which would allow the costs related to the project design and start-up of the trial to be covered while the remainder is mainly paid based on the number of patients recruited and completed as planned. This provides the teams of the sponsor and study sites an incentive to give the necessary priority to these non-commercial studies. Final payments, typically of 5% each, are made when the study report is finalised and the draft manuscript is submitted for publication, thus stimulating the publication of all trials. The funder expects that the personnel capacity planned in the budget tool is made available for the performance of the trial. Any significant deviations need approval by the funder.

The contract between funder and applicant will also detail the possibility of financial audits, allowing the funder to check if the funding received is used for the benefit of the trial.

## Discussion

Clinical trials are expensive to perform, and care should be taken to ensure a qualitative and efficient trial conduct. Key elements for success of publicly funded trials are a solid selection process, a professional conduct of the trial and the swift implementation of the findings in routine care [1, 8]. Trials, including non-commercial trials, are often hampered by a delay in recruitment, sometimes leading to a premature and non-conclusive end [33]. Return on investment is highly sensitive to timelines. Therefore, it is key to develop the right setting and incentives for a timely completion of non-commercial clinical trials. This includes the provision of an adequate budget and a milestone-based payment schedule that stimulates adherence to timelines.

The example presented illustrates a 400-patient, 20-site multicentre comparative effectiveness trial in Belgium. In this example two assumptions are used: first, the study intervention (drug or device) is reimbursed, freely provided or can be purchased at a reasonable cost and, second, the patient study visits and procedures are aligned with routine care.

Table 2 illustrates that project management accounts for a significant proportion of the sponsor-related costs. The budget tool allows one, for example, to model the increase in cost due to a prolonged recruitment period versus the additional cost of opening an additional site for patient recruitment. In most cases, opening an additional site will be more efficient.

The budget tool was successfully adapted for the overall budget calculation of multinational trials funded both by KCE and ZonMw, as well as for the calculation of the national budget needs in the case of Belgian participation in large international trials with an out-of-country sponsor. Anecdotal reports from academic centres in Belgium confirm the uptake of the tool to develop a trial budget for non-KCE funding streams, illustrating the need for such a tool.

The costing tool will need to be adapted when randomised trials based on registries or electronic health records become a reality, possibly leading to dramatic cost reductions [22, 23, 34–37].

A limitation of the tool is that it was designed for use in pragmatic, multicentre trials in Belgium. For pre-market development trials and to some extent for repurposing trials, the tool needs further adaptations, as one may have to add, among other things, study-specific clinical procedures to assess safety and efficacy, coding of all concomitant medication and adverse events, DSMB meetings and central lab testing and readings.

Finally, whereas country-specific pay scales can easily be included in the tool, the tool may need further adaptions based on how trials are organised and delivered in a particular country, e.g. taking into account the presence of structural funding of clinical trial units and of study nurses in hospitals, as is the case in England. In this case, only excess treatment costs and participating site research costs are typically captured [38].

Compared with dedicated clinical trial departments in large pharmaceutical or medical device companies, academic groups have less expertise in preparing the trial tasks and the associated budget [19]. Clinical trial units in universities or large hospitals may fill this gap, assisting researchers with the design, budgeting, conduct and monitoring of clinical trials. Such clinical trial unit networks received public funding to start up in Germany [20] or continue to receive public funding in England [1].

In Belgium, however, clinical trial units in university hospitals mainly focus on legal support and financial oversight for studies where they are a participating site. Building a budget for a clinical trial as a sponsor is an exercise to which they are less accustomed. The set-up of the budget is often taken care of by the specific departments when they apply for funding to be able to maintain their research activities. Most funders either have a maximum amount to spend on research or they base the budget allocated on the number of FTEs involved. Using the activity-based KCE Trials budget tool was new for the teams at the clinical trial units. Some have stated they are now also using the tool to calculate the costs involved for other large studies where they apply for regional or European funding.

In addition to the overall trial budget, the way funds are released during a trial has also been reported to be a determinant of successful delivery. In line with industry practice, a fee-for-performance principle, accomplished through reimbursement for completed CRFs, allows public funders to more easily cope with delays in recruitment or the need to open additional sites in a large trial [39].

More than 12 multicentre randomised trials have been funded in Belgium so far under the KCE Trials programme at a median cost of nearly €2 million, excluding any value-added tax (VAT) per trial [16]. The interventions studied range from medicinal products and medical devices to diets and psychotherapy. In all cases the budget tool proved to be of use as a starting point for the budget negotiation. The first trials are being analysed and reported, but it is too early to judge whether the funding method used was fully appropriate and resulted in a high proportion of successful deliveries. Meanwhile, the tool remains a work in progress and will require some fine-tuning for specific trial settings or new regulatory requirements.

## Conclusions

The budget tool as discussed in this article is a work in progress that allows KCE as a funder to have a transparent, consistent and fair approach when allocating budget to a trial. In addition, it ensures the sponsor has not forgotten to budget any key trial activity. We believe that this funding method results in sufficient trial funding, one of the key elements for a successful delivery.

## Supplementary information

**Additional file 1.** Detailed example of the costs of a trial. Calculation of a study budget with separate columns detailing the costs per item, the margin and the overhead (if applicable).

## Data Availability

Data sharing is not applicable to this article, as no datasets were generated or analysed during the current study. The budget tool and guidance is freely available on the KCE Trials website.

## Abbreviations

CSR: Clinical study report
DBL: Database lock
eCRF: Electronic case report form
FPI: First patient in
FTE: Full-time equivalent
GP: General practice
ICH-GCP: International Conference on Harmonisation-Good Clinical Practice
IMP: Investigational medicinal product
KCE: Belgian Healthcare Knowledge Centre
LPI: Last patient in
LPLV: Last patient last visit
NIH: National Institutes of Health
NIHR: National Institute for Health Research
RCT: Randomised controlled trial
SUSAR: Suspected unexpected serious adverse reaction
TMF: Trial master file
US: United States
