# Supplementary material for: Budgeting of non-commercial clinical trials: development of a budget tool by a public funding agency
Source: Trials. 2019 Dec 11;20:714. doi: 10.1186/s13063-019-3900-8 (PMC6907219; doi:10.1186/s13063-019-3900-8)
Supplement: Supplementary file 1 — Additional file 1. Detailed example of the costs of a trial. Calculation of a study budget with separate columns detailing the costs per item, the margin and the overhead (if applicable). [file 13063_2019_3900_MOESM1_ESM.docx]

Additional file 1:

Table S1: Roles involved in the study team and hourly fees

| **Position** | **Hourly rate** | **Hourly rate including margin and overhead** |
| --- | --- | --- |
| Project Manager | € 50,00 | € 67,28 |
| Clinical Research Associate (monitor) | € 50,00 | € 67,28 |
| Chief Investigator | € 100,00 | € 134,55 |
| Clinical Trial Assistant | € 35,00 | € 47,09 |
| Auditor | € 55,00 | € 74,00 |
| Statistician | € 60,00 | € 80,73 |
| Data Manager | € 50,00 | € 67,28 |
| Study Nurse | € 40,00 | € 53,82 |
| Principal Investigator | € 100,00 | € 134,55 |
| Pharmacist | € 60,00 | € 80,73 |

Table S2: More detailed example of a trial budget based on the use of the budget tool (version 4.0)

|  | Cost per unit | Overall cost of trial | Including margin & overhead |
| --- | --- | --- | --- |
| ***Project design and set-up*** | ***84 825 €*** | | |
| Development of budget and study protocol (including amendments, translation of consent form and any questionnaires), risk assessment and management plan, contracting with funder and vendors, initial selection of sites/GPs | 40 000 € | 40 000 € | 46 800 € |
| On-site feasibility check (per site, for 50% of the sites) | 1 000 € | 10 000 € | 11 700 € |
| Remote feasibility check (for other sites) | 250 € | 2 500 € | 2 925 € |
| Negotiation of site agreement for hospital | 1 000 € | 19 000 € | 23 400 € |
| Subcontracting with tender | 1 000 € | 1 000 € | 1 170 € |
| ***Regulatory, ethics, insurance*** | ***29 835 €*** | | |
| Submission to the leading ethics committee | 2 000 € | 2 000 € | 2 340 € |
| Submission to the ethics committees of participating sites | 500 € | 9 500 € | 11 115 € |
| Communication with ethics committee per study year | 2 000 € | 8 000 € | 9 360 € |
| Submission to regulatory authorities (if applicable) | 2 000 € | 2 000 € | 2 340 € |
| Communication with regulatory authorities per study year | 1 000 € | 4 000 € | 4 680 € |
| ***Study monitoring*** | ***266 079 €*** | | |
| Development of a monitoring plan, risk based. | 1 600 € | 1 600 € | 1 872 € |
| Study monitor familiarisation with trial | 800 € | 1 600 € | 2 153 € |
| On-site initiation visit, monitoring visit, closure visit *(12 to 16 hrs for monitoring in hospital, 8 hrs for GP)* | 800 € | 112 000 € | 150 696 € |
| Fixed average transportation fee per on-site visit | 65 € | 9 100 € | 9 100 € |
| Remote monitoring visits *(4 hrs every two month if no on-site visit)* | 200 € | 76 000 € | 102 258 € |
| ***Quality assurance*** | ***7 020 €*** | | |
| Sponsor audit on-site 2 days by independent auditor, includes visit, preparation and report | 6 000 € | 6 000 € | 7 020 € |
| ***Trial master file (TMF) & administration*** | **66 234 €** | | |
| Set up of (electronic) TMF | 1 000 € | 1 000 € | 1 170 € |
| Set up of investigator files, 4 hrs of clinical trial assistant per site | 140 € | 2 800 € | 3 767 € |
| Maintaining files, 12 hrs of clinical trial assistant per site per year | 420 € | 33 600 € | 45 209 € |
| Sponsor making payments to the sites, 1 per site per year | 150 € | 12 000 € | 14 040 € |
| Archiving, 2 large boxes plus 1 per 4 sites for 25 yrs | 250 € | 1 750 € | 2 048 € |
| ***Safety monitoring and expedited reporting*** | **14 040 €** | | |
| SUSAR documentation and expedited reporting | 400 € | 2 000 € | 2 340 € |
| Data safety monitoring board (DSMB) meeting (1/yr - assuming it is required in this trial) | 2 500 € | 10 000 € | 11 700 € |
| ***Data management*** | **141 570 €** | | |
| Data management plan, database and eCRF set-up and use for comparative effectiveness trial | 75 000 € | 75 000 € | 87 750 € |
| Import, validation, coding, query management per patient in comparative effectiveness trial (*2 hr per patient)* | 50 € | 40 000 € | 53 820 € |
| ***Statistics, report and publication*** | **58 500 €** | | |
| Statistical analysis plan, randomisation plan, programming | 10 000 € | 10 000 € | 11 700 € |
| Statistical analysis and clinical study report | 30 000 € | 30 000 € | 35 100 € |
| Publication, including open access fee | 10 000 € | 10 000 € | 11 700 € |
| ***Project management*** | **367 497 €** | | |
| Trial management group meeting every 2 weeks until end of recruitment and every month thereafter. | 500 € | 33 000 € | 38 610 € |
| Trial steering committee meeting every 4 months during first year, then every 6 months | 2 000 € | 18 000 € | 21 060 € |
| Investigators meeting, at study start and end | 3 500 € | 3 500 € | 4 095 € |
| Investigators meeting, any additional | 2 000 € | 2 000 € | 2 340 € |
| Project manager time per year: 0,2 FTE + 0,025FTE per site | 50 € | 224 000 € | 301 392 € |
| ***Patient and public involvement*** | ***6 552 €*** | | |
| In study design | 2 000 € | 2 000 € | 2 340 € |
| Participation of 2 representatives in steering committee meeting | 200 € | 3 600 € | 4 212 € |
| ***Study Intervention/IMP handling*** | ***152 340 €*** | | |
| Purchase of intervention/IMP/placebo, blinding, packaging, labelling, recovery and destruction of unused product | 130 000 € | 130 000 € | 130 000 € |
| Storage and distribution | 20 000 € | 20 000 € | 20 000 € |
| Central randomisation system and unblinding if applicable | 2 000 € | 2 000 € | 2 340 € |
| ***Site costs*** | ***518 310 €*** | | |
| Start-up fee | 1 000 € | 20 000 € | 23 400 € |
| Per patient visit 2hrs study nurse and 10 min clinician time + 30min clinician time at first visit | 100 € | 300 000 € | 403 650 € |
| lab sample whole blood at 5 visits for central lab | 20 € | 40 000 € | 53 820 € |
| Local pharmacy costs for transfer study | 1 350 € | 27 000 € | 31 590 € |
| Archiving box for 25 yrs | 250 € | 5 000 € | 5 850 € |
| ***External vendors*** | ***0 €*** | | |
| Study specific equipment or external services | included in IMP budget | | |
|  | | | |
| *TOTAL BUDGET without VAT* | 1 725 672 € | | |
| *TOTAL BUDGET with 21% VAT* | 2 088 063 € | | |

*To calculate the budget for this study, we took into account the following study parameters: 20 sites, 400 patients, 7 visits per patient, recruitment period of 18 months, treatment period of 24 months, total study duration of 4 year, 5 on-site monitoring visits and every 2 months a remote monitoring visit, 3^rd^ party placebo development, packaging, labelling, distribution*
